# Early Cytokine Response to Infection with Pathogenic vs Non-Pathogenic Organisms in a Mouse Model of Endodontic Infection

**DOI:** 10.1371/journal.pone.0132752

**Published:** 2015-07-14

**Authors:** Aritsune Matsui, Danielle Stephens, Alpdogan Kantarci, Susan R. Rittling

**Affiliations:** 1 The Forsyth Institute, Cambridge, Massachusetts, United States of America; 2 Harvard School of Dental Medicine, Boston, Massachusetts, United States of America; University of Pittsburgh, UNITED STATES

## Abstract

Using the subcutaneous chamber model of infection, we showed previously that a mixture of four endodontic pathogens (EP: *P*. *intermedia*, *F*. *nucleatum*, *S*. *intermedius* and *P*. *micra*) are able to persist without clearance for up to seven days, while a non-pathogenic oral species, *S*. *mitis*, was substantially cleared in this time. Here we have compared the cytokine response inside the chambers against these microorganisms. A majority of cytokines tested (17/24) showed different patterns of expression. Several cytokines had a peak of expression at 2 h after infection in response to the EP, while none showed this pattern in *S*. *mitis* infections. Chemokines were uniformly present at similar or higher levels in response to *S*. *mitis*, with redundant expression of CXCR2 ligands, while several growth/survival factors were present at higher levels in EP infections. Protease activity expressed by EP may be responsible for the lower levels of some chemokines. T-cell associated cytokines were in general expressed at extremely low levels, and did not differ between the two infections. The inflammatory markers IL-6, IL-1α and IL1-β were expressed at similar levels in both infections at early times, while TNFα was preferentially present in *S*. *mitis* infections. In EP infected chambers, reciprocal changes in levels of IL-6 and IL-1α were observed at later times suggesting a switch in the inflammatory response. Analysis of the cytokine response to infection with the individual species from the EP mix suggests that *P*. *intermedia* drives this inflammatory switch. Together these results show a surprising level of divergence of the host response to pathogenic and non-pathogenic organisms associated with oral infections, and supports a dominant effect of *P*. *intermedia* in polymicrobial endodontic infections.

## Introduction

Endodontic infections (also known as apical periodontitis) are always polymicrobial [1], and can induce an intense host inflammatory response leading to pulpitis and necrosis [2]. Endodontic diseases are common, with lifetime prevalence estimates as high as 20–100% infections per individual [3]. The innate immune response to microbial species involved in the etiology of endodontic diseases principally involves neutrophils, the primary defense against bacterial pathogens. The mechanisms of the host response to these infectious agents have been elucidated through the use of rodent models. In these models of endodontic infection, the pulp chamber of mouse molar teeth are typically exposed and inoculated with bacterial species associated with human endodontic infections [4]. The use of genetically engineered mice has been especially useful in understanding the role of many inflammatory factors [5–9]. However, in these mouse models, the amount of infected tissue is quite small, and determination of cytokine levels by ELISA can be technically challenging. In some cases, protein measurement with ELISA has been reported, while in others the levels of cytokine production were estimated by qPCR for the respective mRNAs. Regardless of the method of detection, the number of cytokines assayed has been relatively small and higher throughput analysis techniques are needed.

In order to overcome these limitations, we adapted the mouse subcutaneous chamber model [10] for a detailed analysis of the host response to endodontic infection. We hypothesize that this model mimics in many ways infection within the pulp chamber, especially in the incorporation of a solid tubular surface within which the infecting organisms are inoculated. Using this model, we discovered that endodontic species associated with human endodontic infections, particularly *Prevotella intermedia*, disrupt the host response by damaging or killing the infiltrating neutrophils, preventing them from carrying out anti-bacterial functions [11]. On the other hand, a non-pathogenic species, *Streptococcus mitis*, is efficiently cleared in this chamber model. This model also provides an ideal system for the analysis of the cytokine/chemokine response to infection with endodontic organisms, especially at early times (up to 7 days) after infection [12]. The chamber fluid is easily recovered and provides ample material for the analysis of the level of a number of secretory products. Here, we report the results of these analyses, comparing the cytokine response to the pathogenic and non-pathogenic organisms. In particular, we report that there is redundancy in the expression of ELR+ chemokines, with MIP-2 being the major CXCR2 ligand produced in infections with the pathogenic species. Th1 cytokines are present at extremely low levels, and are unlikely to play a predominant role in these early infections. The inflammatory mediators IL-6 and IL-1α are present in a reciprocal relationship in pathogen infected chambers, supporting a switch from an IL-6 to an IL-1α based response at later times. These results add to our understanding of the early host response to infection with endodontic pathogens, and highlight ways in which this response is modified by the infecting species.

## Materials and Methods

### Mice

Male C57BL/6 mice were purchased from Jackson Laboratories and used at 6–8 weeks of age. All mice were maintained in ventilated racks under SPF conditions on a 12 h light-dark cycle, and were housed in groups of 3–5 with ad libitum access to food and chlorinated water. Titanium chambers, 1.5 x 0.5 cm tightly wound coils of 0.037” diameter wire, were implanted under the dorsal skin of anesthetized mice, two coils per mouse as previously described [11]. Ten days after implantation, the chambers were infected with 1 x 10^9^ CFU of bacteria, either individual species or an equal mixture of the four pathogenic species as indicated. Chamber contents were recovered at different times after infection by flushing the chambers with 0.7 ml of PBS with 5% FBS after euthanasia of the mice by CO_2_ inhalation as recommended by the AVMA. Contents of both chambers were pooled. The 0 h time point represents uninfected animals. The results presented are a secondary analysis of samples from previously published work [11]. Ethics: All experiments with mice were approved by the Forsyth Institutional Animal Care and Use Committee under protocol # 11–009. The Forsyth Animal Program is fully accredited by AAALAC.

### Bacterial preparations

Bacterial species used for infection were all obtained from ATCC and maintained as frozen stocks at Forsyth. Endodontic pathogens (EP) included *Prevotella intermedia* (ATCC 25611), *Streptococcus intermedius* (ATCC 27335), *Fusobacterium nucleatum* (ATCC 25586), and *Parvimonas micra* (ATCC 33270): these species were grown on Trypticase Soy Agar with 5% sheep blood (TSA II plate, BBL) under anaerobic conditions (80% N_2_, 10% H_2_ and 10% CO_2_). *Streptococcus mitis* was grown in Todd-Hewitt broth. Bacteria for injection were prepared as described previously [11]. For chamber infection, individual species, mixtures of the four species, or *S*. *mitis* were diluted into PBS at 1x10^9^ CFU /100μl.

### Cytokine Analyses

Chamber fluid collected from two chambers per mouse was pooled and centrifuged at 240 x g for 5 min at 4°C, and the supernatant stored at -80°C. Prior to analysis, protease inhibitor cocktail (Roche) was added according to the manufacturer’s instructions. Fifty microliters of each supernatant was used for analysis of 24 cytokines and chemokines using a custom mouse 24-plex panel (Millipore, MCytoMag 70K) according to the manufacturer’s instructions. For analysis of cytokine induction by individual species, a custom mouse 5-plex panel was used. Samples were analyzed using a Bio-Plex 200 apparatus, and concentrations determined from standard curves run in parallel. Cytokine concentrations obtained from these analyses were corrected for dilution during the collection process, and are reported as the estimated original concentrations in the chambers. Each sample was in a single well.

### Protease Assay

Cysteine proteases were assayed using the fluorescent substrate BOC-Val-Leu-Lys-AMC (Bachem). Reactions included chamber fluid and 0.25 mM substrate in 0.1M Tris pH 7.5/50 mM EDTA. Roche Complete Mini Protease Inhibitor (EDTA-free) was added to 1x where indicated. Reactions were performed in black plates at 37°C in a BMC Optima spectrophotometer; fluorescence was determined at 460 nm every minute for 1 h. The slope of the curve in the linear portion was determined using the Optima software.

### Western Blot

KC (Peprotech, 25 ng/reaction) was incubated with chamber fluid at 37°C for 14 h with or without Roche Protease Inhibitor as indicated. 2 x SDS PAGE sample buffer was added and samples were separated on 4–20% Tris-MOPS gels (Genscript), and transferred to PVDF membranes. KC was identified using a biotinylated rabbit anti-mouse KC antibody (Peprotech) followed by streptavidin peroxidase (Roche). Three samples from individual mice were analyzed in parallel.

### Statistical Analysis

Data are presented as mean +/- SEM. For EP and *S*. *mitis* infections, n = 6 for the 0 h time point, 9 for 2, 12 and 24 h and 5 for 72 and 168 h (7 days). For time courses, data are plotted at equal intervals along the x-axis for clarity. Statistical significance between groups at each time point was determined by unpaired t-test with Bonferonni correction for multiple comparisons [13], p < 4 x 10^-4^ was considered significant (***). 1 way ANOVA was used to compare values across different time points using Graph-Pad Prism.

## Results

### Acute cytokine response

**Fig 1:**
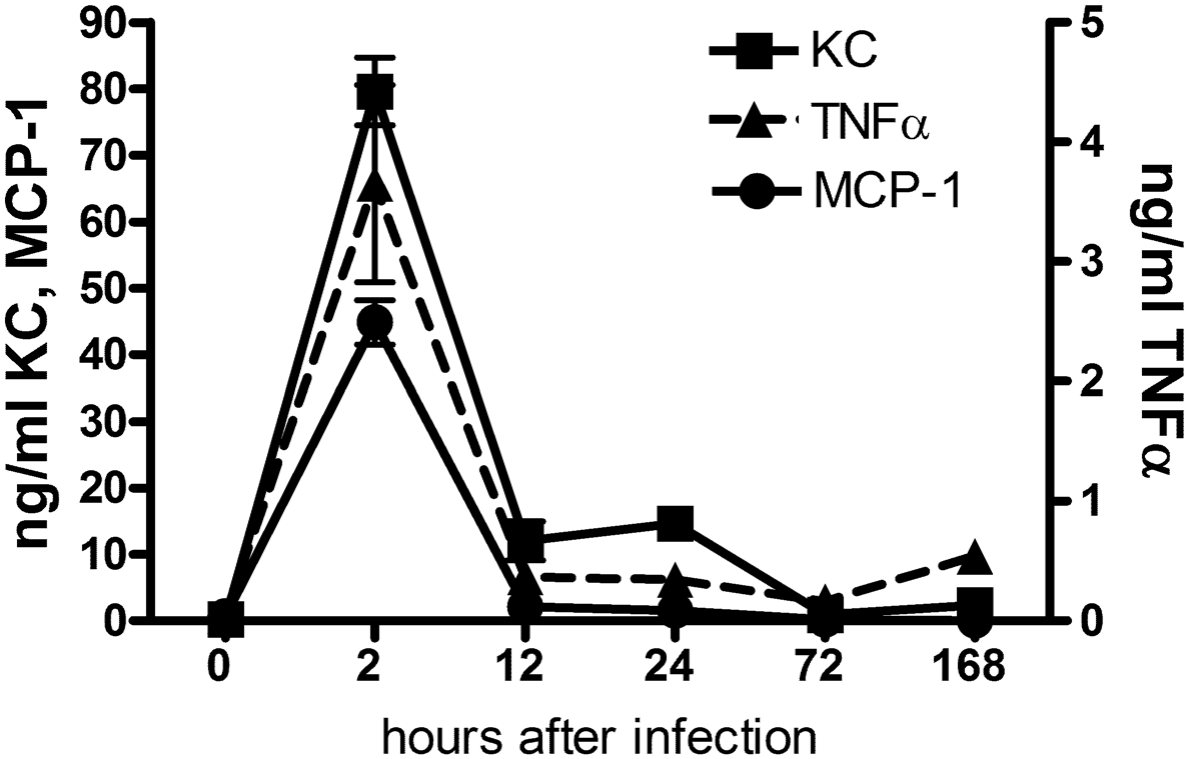
Acute increase in some cytokines immediately after infection. A. Levels of KC and MCP-1 (left axis) and TNFα (right axis) in EP infected chambers at different times after infection. p<0.001 for all analytes 2 vs 12 h.

We compared the cytokine response to two kinds of infection in the subcutaneous mouse chamber model. Chambers were infected with either a mixture of four endodontic pathogens: *P*. *intermedia*, *F*. *nucleatum*, *S*. *intermedius*, and *P*. *micra* (EP), which have been well documented to cause periapical lesions and associated bone loss in mice [7, 9, 14], or a non-pathogenic oral bacterial species *Streptococcus mitis*, a commensal oral organism not strongly associated with oral infections. Chamber fluid was collected at different times after infection, and cytokine expression was determined by Luminex analysis in the presence of protease inhibitors. Cytokine expression levels determined in chamber fluid were corrected for the estimated dilution during collection (about 7-fold) so the values reported are the estimated actual concentrations inside the chamber during the response to infection. A striking difference in the response to these two infections was a strong but transient induction of high levels of many cytokines immediately after infection with the endodontic pathogens. About half the chemokines and cytokines analyzed showed this acute pattern of expression, with peak levels at 2 h after infection, returning to near baseline by 12 h post infection. In *S*. *mitis* infected chambers, in contrast, no cytokines were present at higher levels at 2 h than at 12 h. Fig 1 shows representative results for a subset of analytes displaying this pattern of expression in the EP group: KC (CXCL1), TNFα, and MCP-1. Analytes showing this pattern of expression are referred to as acute response cytokines as compared with those whose expression pattern was more generally upregulated.

Acute response was defined as expression significantly higher (p<0.001) at 2 h than at 12 h. In most subsequent figures, the 2 h time point is omitted for cytokines showing this acute expression pattern. Table 1 summarizes the average expression level of all cytokines tested at 24 h after infection with EP or *S*.*mitis*, and indicates which show an acute response.

**Table 1:** Summary of expression of all cytokines measured.

	expression at 24 hours, ng/ml				
Analyte	EP	Sm	P value^a^	Acute Responder^b^	higher in EP or *S*.*mitis*
Chemokines					
**KC (CXCL1)**	14.68	58.10	8.542E-12	yes	*S*. *mitis*
**MIP-2 (CXCL2)**	77.88	69.92	6.199E-02	yes	similar
**LIX (CXCL5)**	10.42	24.41	3.571E-06	yes	*S*. *mitis*
**MIP-1α (CCL3)**	2.65	10.37	1.434E-07	no	*S*. *mitis*
**MIP-1β (CCL4)**	2.03	11.86	3.748E-04	no	*S*.*mitis*
**Eotaxin (CCL11)**	0.60	1.48	2.778E-07	yes	*S*.*mitis*
**IP-10 (CXCL10)**	0.40	4.86	2.624E-11	yes	*S*.*mitis*
**RANTES (CCL5)**	0.16	0.16	9.442E-01	no	similar
**MCP-1 (CCL2)**	1.56	4.20	3.480E-04	yes	*S*.*mitis*
**MIG (CXCL9)**	0.74	0.67	6.492E-01	no	similar
Cytokines					
**IL-1α**	5.51	3.45	7.826E-03	no	EP, late
**IL-1β**	7.51	9.85	4.148E-02	no	similar
**IL-6**	106.70	100.20	5.304E-03	yes	*S*.*mitis*, late
**TNFα**	0.34	1.82	7.423E-09	yes	*S*.*mitis*
**IL-2**	0.013	0.008	7.612E-02	no	similar
**IL-5**	0.00	0.00	7.401E-02	yes	similar
**IL-10**	0.44	1.75	1.724E-04	yes	*S*.*mitis*
**IL-12p70**	0.01	0.01	8.398E-01	yes	similar
**IFN-γ**	0.14	0.05	1.309E-04	no	EP
**IL-17**	0.08	0.09	7.441E-01	yes	similar
Survival Factors					
**G-CSF**	89.03	80.02	7.084E-05	no	similar
**M-CSF**	10.19	0.08	2.725E-11	no	EP
**LIF**	6.89	2.78	4.227E-06	no	EP
**VEGF**	3.21	8.04	1.735E-06	no	*S*.*mitis*

^a)^ P value (EP vs *S*. *mitis*) determined by unpaired t-test.

^b)^ Acute responder cytokines are defined as those expressed significantly (p<0.001) higher at 2 h than at 12 h after infection. EP- endodontic pathogens, Sm–*S*. *mitis*.

### CXCR2 ligands

**Fig 2:**
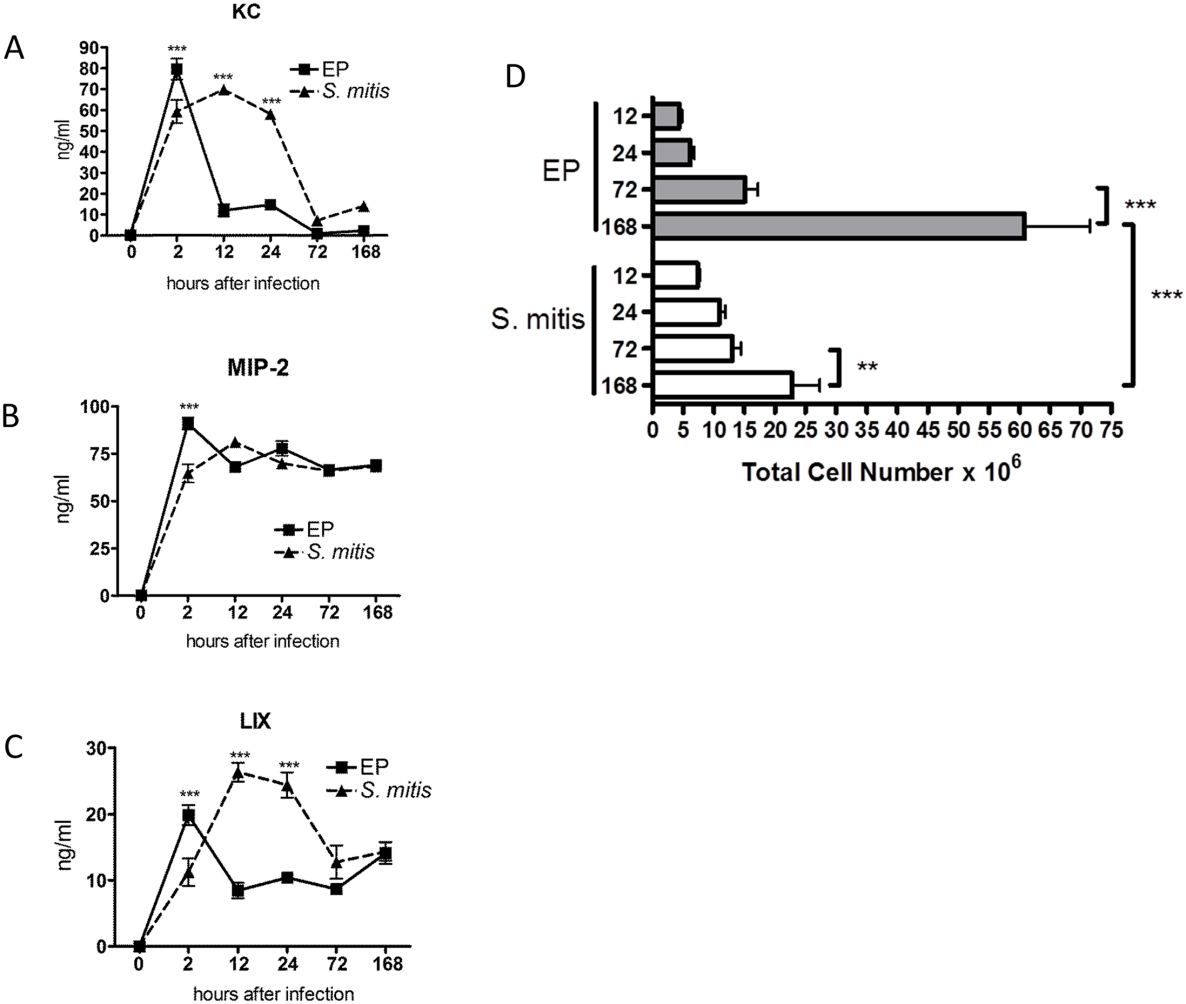
CXCR2 ligands are expressed in infected chambers. Levels of CXCR2 ligands KC (A), MIP-2 (B), and LIX (C) in chamber fluid from EP and S. mitis infected chambers at different times after infection. * - p< 4x 10–4 for EP vs S. mitis as determined by unpaired t-test. (D) Total cell numbers recovered from chambers at different times after infection (Y-axis) with the species as indicated. ***, p<0.001; **, p<0.01 by unpaired t-test.

Physiologically relevant concentrations (50–200 ng/ml) of the three CXCR2 ELR+ chemokine ligands expressed in mouse (KC, MIP-2 and LIX) were measured in chamber fluids (Fig 2A–2C). Only MIP-2 was expressed at similar levels in response to both infections. Furthermore the total level of MIP-2 was maintained at a constant level throughout the duration of the infection. This was the case in both the EP and *S*. *mitis* infected chambers, consistent with the continued presence of neutrophils in these chambers even at later time points (Fig 2D). However, the other two ELR+ chemokines, KC and LIX, were found at much lower levels in EP infected chambers as compared to *S*. *mitis*. Note that both these chemokines show the acute response as described in Fig 1. This observation suggests that MIP-2 alone is sufficient for neutrophil recruitment, since neutrophil levels were similar in response to either infection [11]. There was a significant increase in total cell numbers (predominantly neutrophils) at 168 h in both chambers, and this increase was significantly higher in the EP infected chambers than in *S*. *mitis* infection (Fig 2D) [11]. However, this was not reflected in increased expression of the CXCR2 ligands, suggesting that an alternative mechanism to recruit neutrophils is employed by 7 days after infection.

**Fig 3:**
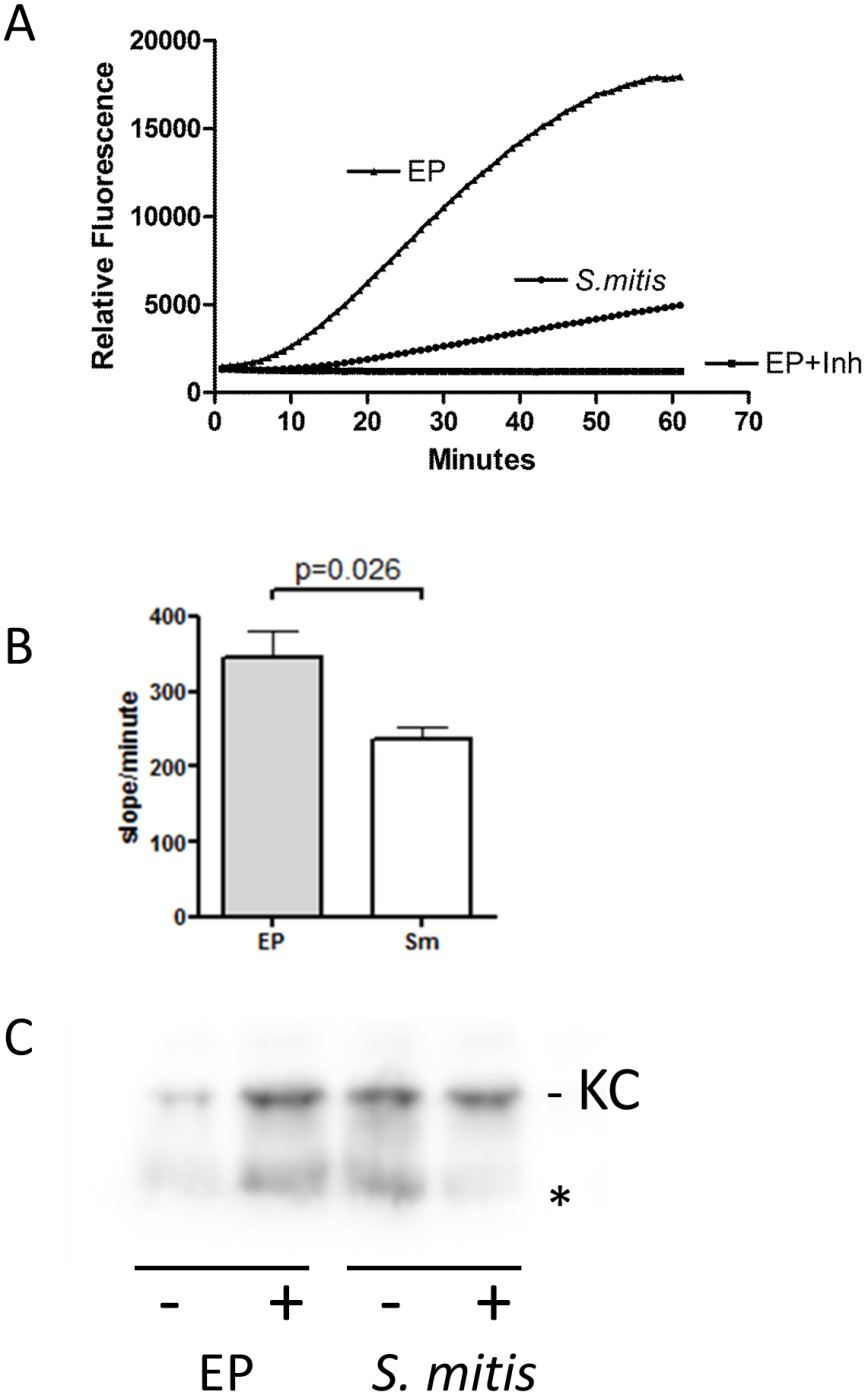
KC is degraded by proteases in the EP infected chambers. (A) Protease activity in chamber fluid from EP or S. mitis infected chambers 24 h after infection. Increased fluorescence results from cleavage of the substrate Val-Leu-Lys-AMC. Inh - reaction performed in the presence of protease inhibitor cocktail. (B) Protease activity (slope of the linear portion of the curve; change in fluorescence/minute) in EP and S. mitis chamber fluid, n = 3 individual mice. (C) Western blot of KC levels after incubation in chamber fluid. 25 ng of KC was added to 10 μl of chamber fluid and incubated for 14 h in the presence or absence of protease inhibitor cocktail (Inh), then separated on a 4–20% SDS Page gel. KC runs at about 15 kD; the lower band (*) is likely a degradation product. Representative results of three independent determinations. Average values (% KC detected without vs with protease inhibitor) were 34.50 ± 6.054 for EP and 119.0 ± 25.95 for S. mitis, p<0.05.

*P*. *intermedia* produces a number of proteases, including a cysteine protease interpain, which can degrade complement [15] and participates in iron acquisition [16]. Protease activity was measured in chamber fluid from EP and *S*. *mitis* infected chambers using the substrate Val-Leu-Lys-AMC: this activity was significantly higher in EP infected chambers at 24 h after infection (Fig 3A and 3B). Val-Leu-Lys is a substrate for interpain, however, the measured activity was not blocked by the cysteine protease inhibitor E64 (data not shown), suggesting that serine proteases, even including host proteases such as plasmin may be responsible for this activity. Therefore, we considered the possibility that the lower levels of KC or LIX in EP infected chambers could be due to proteolytic degradation. EP or *S*. *mitis* chamber fluid was incubated *in vitro* (37°C for 14 h) with purified KC with or without protease inhibitor, and the amount of KC remaining determined by western blot. The results indicated that about 65% of KC was degraded/lost in this assay in EP chamber fluid, while more than 100% of the KC was recovered from *S*. *mitis* chamber fluid (p<0.05, n = 3, representative blot shown in Fig 3C), suggesting that proteolytic degradation may be responsible for the lower levels of KC in EP infected chambers, and possibly other cytokines as well.

### Low level expression of cytokines associated with the adaptive immune response

**Fig 4:**
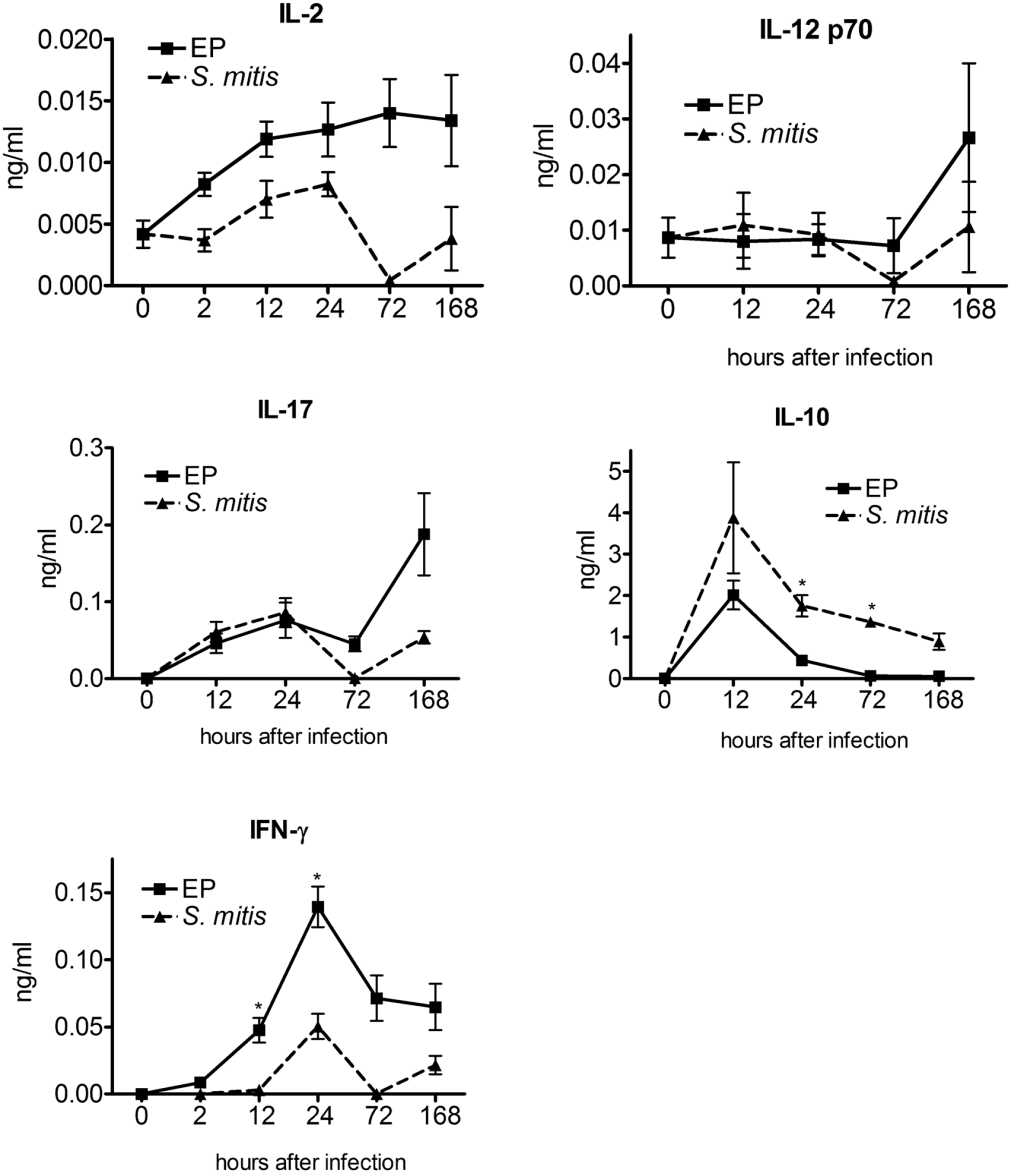
Levels of Th1/Th2 cytokines IL-2, IL-12p70, IL-17, IL-10 and IFN-γ in infected chambers at different times after infection. * - p<4x 10–4 for EP vs S. mitis as determined by unpaired t-test.

Levels of several interleukins (IL-2, IL-12p70, IFNγ, IL-17) associated with Th1 and Th17 responses were also examined in these chamber fluids (Fig 4). These were expressed at very low (sub-nanogram/ml) levels, and in some cases (IL-12p70 and IL-17) expression was not significantly increased above the uninfected control level at most time points. Interferon-γ was expressed at relatively high levels, and was significantly higher in EP infected chambers than in *S*. *mitis* at 24 and 72 h. IL-10 was expressed at higher levels (up to 4 ng/ml), but in a transient manner, with a peak of expression at 12 h, returning to baseline thereafter.

### Expression of inflammatory mediators

**Fig 5:**
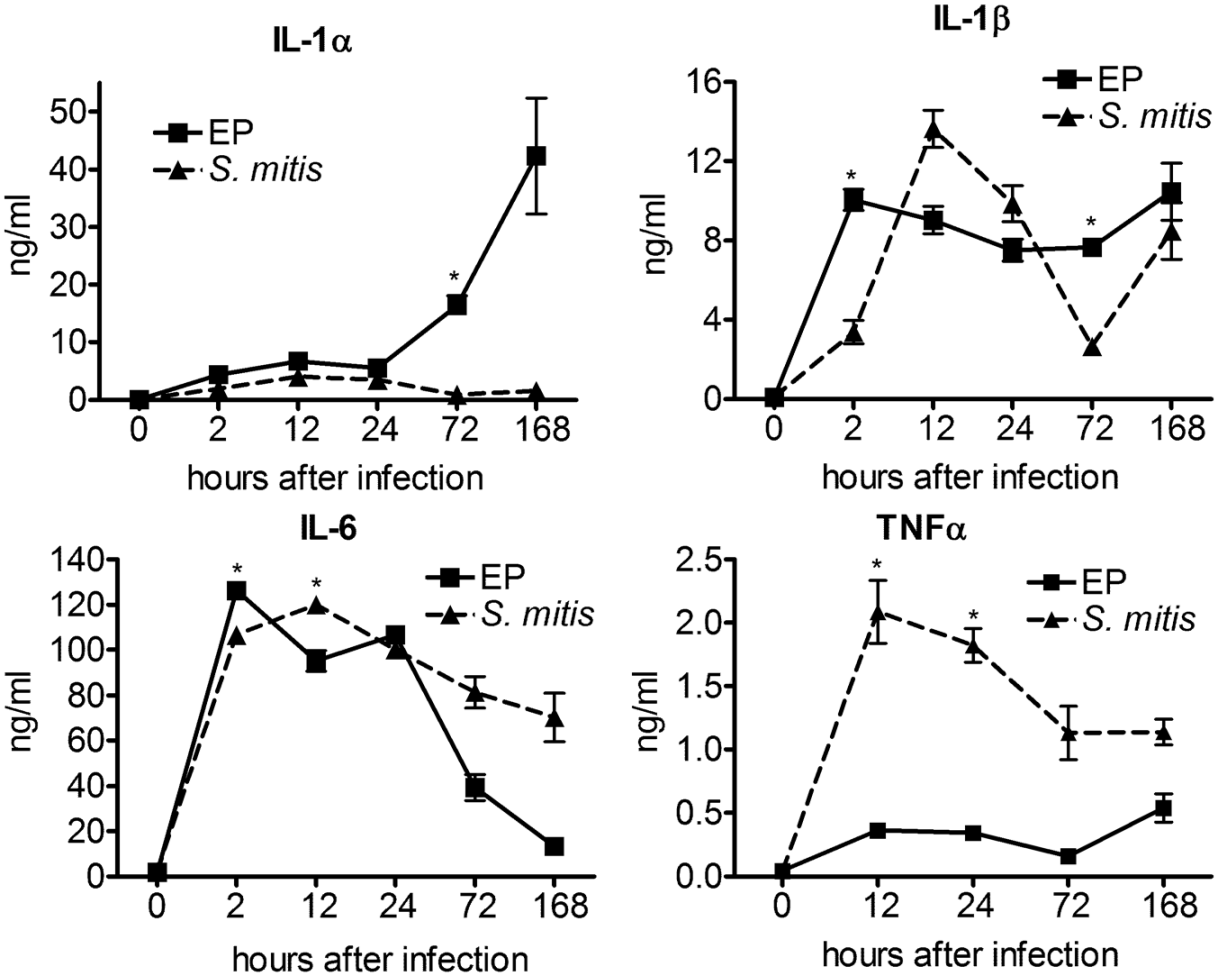
Levels of inflammatory mediators IL-1α, IL-1β, IL-6 and TNFα. Cytokines were measured in chamber fluids collected at different times after infection as indicated. * - p<4x 10–4, for EP vs S. mitis as determined by unpaired t-test. TNFα, on the other hand, was present at much higher levels in *S*. *mitis* infected chambers as compared to EP, particularly at 12 and 24 h after infection. Levels of this cytokine remained very low in EP infected chambers at all time points except for 2 h (Fig 1). In this case, *in vitro* analysis showed that TNFα levels were not reduced after incubation in EP chamber fluid (data not shown), suggesting that protease degradation is not responsible for the low levels of TNFα in EP infected chambers.

Expression of the inflammatory mediators IL-1α, IL-1β, and IL-6 was similar in EP and *S*. *mitis* infected chambers at early times (2–24 h): even though there were significant differences at some time points. The peak of IL-1β expression was at 2 h in EP infection, but not until 12 h in *S*. *mitis* infection, but over the course of infection, the levels of this cytokine were similar in both cases. (Fig 5). Of interest however is the increase in IL-1α expression at later times in response to EP, but not *S*. *mitis* infection. As described previously, infections in the EP chambers do not resolve, and by 168 h, swelling and pus formation was observed [11]. This coincides with a 6-fold increase in IL-1α as compared to the level at 12 h after infection. This observation suggests that IL-1α at least partially mediates the escalation in inflammation observed 7 days after infection with EP organisms, which are unable to be cleared by the host innate immune system. Interestingly, this increase in IL-1α is accompanied by a decrease in IL-6 expression, suggesting a shift in the nature of the response to these infections at later times. By contrast, the levels of expression of these cytokines are maintained at relatively constant levels in the *S*. *mitis* infected chambers. In *S*. *mitis* infection, the bacterial load has decreased by nearly five logs by 7 days, but remains above 10^4^ CFU/ml[11], so the antibacterial inflammatory program is probably still in place in these chambers at 7 days after infection.

### Survival Factors

**Fig 6:**
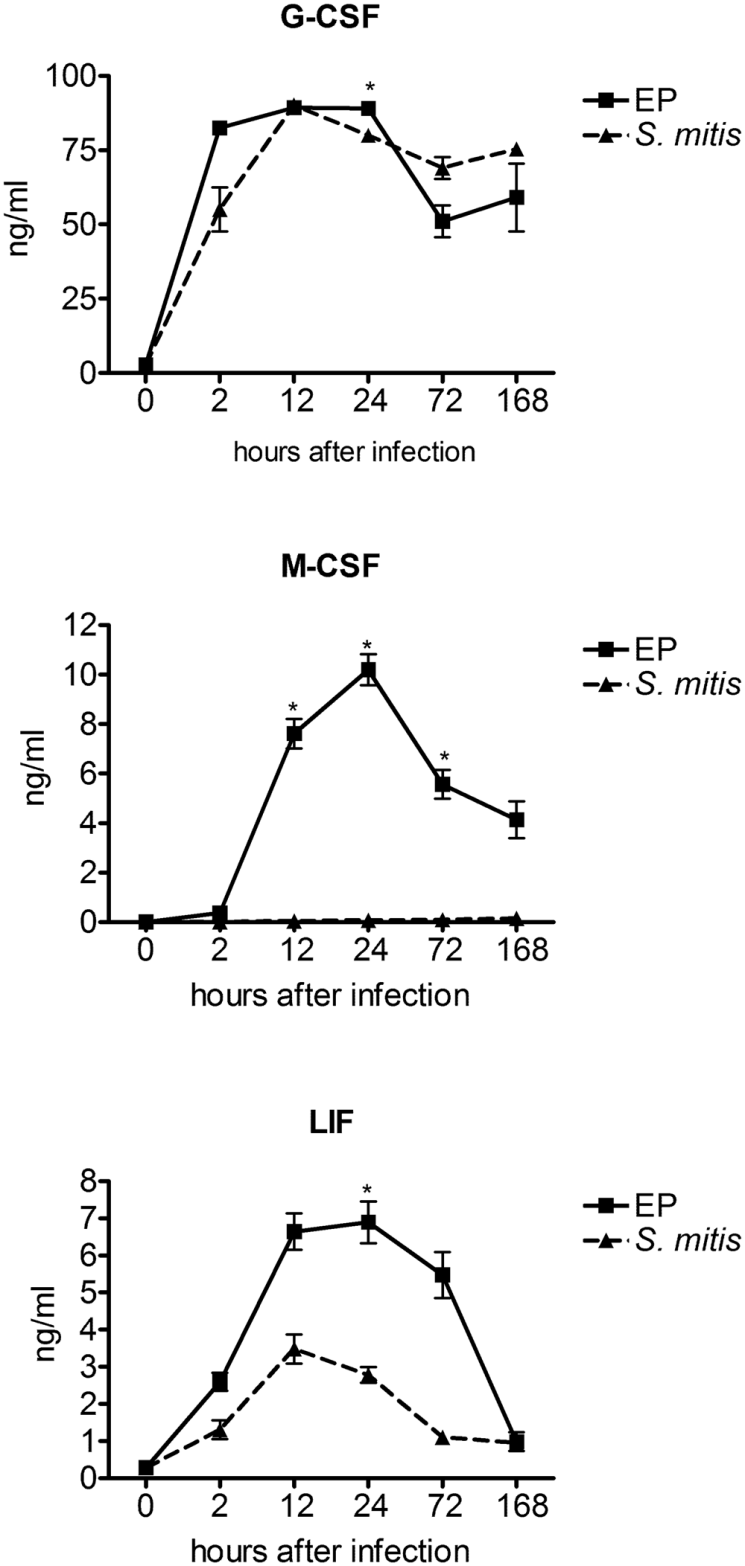
Levels of survival factors. G-CSF (top panel) M-CSF (middle panel) and LIF (bottom panel) were measured in chamber fluid collected at different times after infection as indicated. * - p<4 x 10–4 for EP vs S. mitis as determined by unpaired t-test.

Several cytokines whose main function is to promote survival of various cell types were present at physiologically relevant levels in the chamber fluid. G-CSF was expressed at very high levels in both EP and *S*. *mitis* infected chambers at all time points, consistent with the need to support neutrophil function in both types of chambers (Fig 6). Of interest, M-CSF and LIF were present at much higher levels in EP infected chambers than in *S*. *mitis* infection. M-CSF is a growth and differentiation factor for monocytes and macrophages, but we were consistently unable to identify these cell types within the chambers. LIF, which was originally identified as a leukocyte inhibitory factor, and is induced by LPS [17], was also present at significantly higher levels in EP infections. Perhaps expression of these cytokines is a response by the innate immune system to the loss of neutrophils to support survival of other cell types such as monocytes in the face of the killing function of the EP bacterial species.

### Cytokine response to infection with individual species

**Fig 7:**
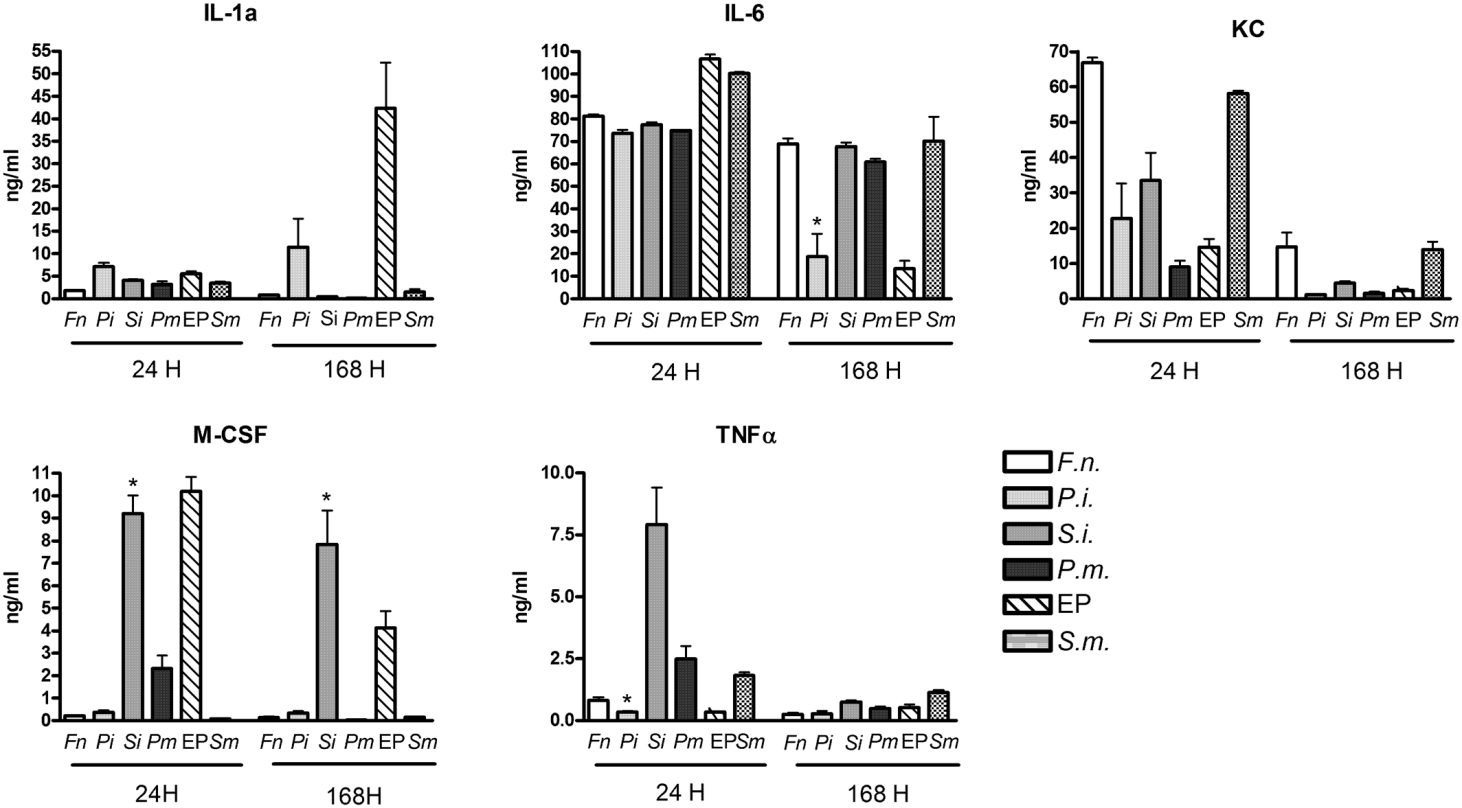
Cytokine levels in chambers infected with individual species. Cytokine levels were determined as previously described in fluid collected from chambers at 24 and 168 h after infection with the individual species as indicated. Data from the EP mixture (EP) and S. mitis (Sm) infections from previous figures are shown for comparison. Individual species are indicated: Fn–*F*. *nucleatum*; Pi—*P*. *intermedia*; Si–*S*. *intermedius*; Pm–*P*. *micra*. N = 4 for all samples, except Fn 24 h (n = 3) and Si 24 h (n = 5). *- p<0.01 compared to the other three individual species.

The large differences in cytokine response to EP and *S*. *mitis* infections were unexpected. Since *S*. *mitis* is Gram positive, and the EP mixture contains both Gram negative and Gram positive species, we were interested to understand if this difference could be partially responsible for the different responses. Accordingly, we tested the host response to infection with the individual species comprising the EP mixture (*F*. *nucleatum*, *P*. *intermedia*, *S*. *intermedius*, *or P*. *micra*). Levels of cytokines that showed large differences between EP and *S*. *mitis* (IL-1α, IL-6, KC, M-CSF and TNFα) were assayed in chamber fluid collected at 24 and 168 h after infection with the individual species (Fig 7). In all cases, live bacteria were still present at 168 h after infection, at levels intermediate between the EP mix and *S*. *mitis* [11].

There are several observations that can be made from this analysis. First, there is no consistent difference between the response to Gram positive (*S*. *intermedius* and *P*. *micra*) and Gram negative (*F*. *nucleatum* and *P*. *intermedia*) organisms. Second, it appears that *P*. *intermedia* is able to drive the cytokine response to IL-1α, IL-6 and TNFα, since the cytokine response to *Pi* most closely matches that of the EP mix at 168 h (Il-1α and IL-6) or 24 h (TNFα). Third, the response to KC seems to be specific to each organism, with the highest response to *F*. *nucleatum*. And finally, *S*. *intermedius* appears to be responsible for the high level of M-CSF induced by the EP mix.

## Discussion

Here we have used cytokine expression as a measure of the extent and nature of the host immune response to oral pathogenic and non-pathogenic bacteria. We have taken advantage of the unique features of the chamber model of infection to perform a comprehensive analysis of the cytokine and chemokine response to these species. In this model, the chambers contain bacteria and infiltrating cells, mostly neutrophils, and the cytokine content of the fluid recovered from these chambers is easily analyzed. In total, the response to these two types of organisms was very different, with only 37% of the analytes examined (9/24) showing a similar pattern of expression between the two infections. This may partially be because *S*. *mitis* is a Gram-positive organism, while the EP mix contains both Gram-positive and Gram-negative bacteria. Previous studies have demonstrated that Gram-negative and Gram-positive bacteria elicit different cytokine responses *in vitro* in human monocytes and PBMC [18, 19], with increased expression of more cytokines induced by Gram-positive species (IL-12, IFN-γ, TNF-α and IL-1β) than Gram-negative species (IL-6, IL-10 and IL-8 (the human homolog of KC)). Our results are similar to these in that in many cases (11/24) cytokine expression was higher in Gram-positive *S*. *mitis* infection than in EP infection (both Gram negative and Gram positive), while only 4/24 cytokines were present at higher levels in EP infected chambers, without considering the acute response cytokines. However, analysis of the cytokine response to the individual species including two Gram negative and two Gram positive species did not suggest a widespread differential response. For IL-1α and IL-6 at 24 h, there was a similar response to all the species tested. The Gram positive species did elicit a higher level of M-CSF and TNFα at 24 h but these are the only instances of such a polarization. While we do not know which cell types express the different cytokines in our model, it is likely that dermal epithelial cells and fibroblasts as well as monocytes/macrophages are responsible, which may explain why our results differ from those in PBMC.

Neutrophil accumulation at sites of infection is regulated by several G-protein coupled receptors on the cell surface. Most important among these are FPR1 and FPR2 (in mouse) that bind formylated peptides generated by bacteria [20]. The CXCR2 receptor, which in the mouse binds three chemokines of the ELR+ family [21], is also important for neutrophil accumulation. In mice lacking CXCR2, neutrophils are unable to effectively exit the circulation to access infected tissues, confirming the importance of these receptors in the neutrophil response [22, 23]. It is of interest that all three CXCR2 ligands are expressed in *S*. *mitis* infected chambers at physiological levels, confirming redundancy built into the system. This redundancy is clearly required in the case of the EP infections, where the KC and LIX responses are blunted, but neutrophils still accumulate to nearly the same level as in *S*. *mitis* chambers. One reason for these differences is likely the degradation of KC by protease(s) present in the EP infected chambers (Fig 1E). It is not known if LIX is similarly susceptible to protease digestion in the EP chambers. Furthermore, the level of KC-degrading protease activity expressed by the individual species was not assessed: this may affect the observed variable KC response to each species. Additionally, the cells that produce KC and LIX may be blocked or killed by the EP bacteria, preventing the synthesis of these chemokines.

Total neutrophil numbers remain at a steady level in the *S*. *mitis* infected chambers for 72 h after infection, with a slight increase at 7 days, while the proportion of live neutrophils declines significantly after 24 h [11]. These results suggest that neutrophil clearance may be slow in this system and that neutrophil entry into the chambers is represented by the live neutrophil population seen only at 12 and 24 h. The decline in live neutrophil numbers is coincident with the decrease in the levels of KC and LIX, while MIP-2 levels remain high. These results may suggest that KC and LIX are most responsible for bringing neutrophils to the chamber, but additional experiments are required to determine this. Similarly, the mechanism of the increase in cell numbers in the chambers 168 h after infection is not clear, but seems to be mediated by a mechanism different from the ELR+ chemokines.

The low level of T-cell associated cytokines IL-2, IL-12, and IL-17 are consistent with the lack of effect of the adaptive immune response at early times in these infections [5, 6, 9]. IFN-γ and IL-10 on the other hand are expressed at somewhat higher levels, albeit transiently. IL-10 plays a major role in controlling inflammation in endodontic infection in the mouse, consistent with its higher level of expression [7]. Its peak of expression at 12 h coincides with the peak level of live neutrophils in the *S*. *mitis* chambers, suggesting that an anti-inflammatory response is induced after the initial influx of neutrophils. It is of interest however that IL-10 disappears from the EP infected chambers by 72 h after infection; perhaps it is induced again at even later times after infection. The increase in IL-17 at later times in EP infected chambers could signal the start of an adaptive Th17-based immune response. IL-17 has been shown to have a protective role in endodontic infections mediated by MIP-2 and IL-1β [24], which are also present in the chambers at this time. However, the low level of all these cytokines likely reflects the short time frame of this experiment, during which an adaptive response may not be effectively generated.

The contrasting regulation of IL-6 and IL-1α in the EP infected chambers is of considerable interest. Both of these cytokines were shown in rat or mouse models of endodontic infection to have a protective effect, although the role of IL-1α appeared more central [25–27]. In other models of infection, IL-1 signaling has been shown to be a critical regulator of chemokine expression and neutrophil accumulation [28]. It is of interest, therefore that expression of IL-1α is relatively low at early times, increasing more than 6 fold in EP infected chambers at later times of infection, at the same time as IL-6 expression is being reduced. These results suggest that there may be a switch in the inflammatory program at later times after infection, from an IL-6 based response to a response mediated primarily by IL-1α. Since IL-1α is not induced during the response to *S*. *mitis*, this suggests that the IL-1α response is required when the standard IL-6 response is insufficient. Results with the individual species suggest that *P*. *intermedia* drives both the increased IL-1α expression as well as the decrease in IL-6 at 168 h, which is consistent with our hypothesis that this species is responsible for disabling the initial neutrophil response to these infections, triggering the secondary IL-1α response. A similar pattern of expression of IL-6 peaking at early times and IL-1α peaking later was also observed in periapical lesions of mice following pulp exposure [29], further confirming that the chamber model replicates the response in the pulp chamber.

Cytokines with roles in promoting survival of various cell types are highly expressed in the EP infected chambers. G-CSF, which mediates neutrophil production in the bone marrow, is present at very high levels in both types of chambers and at all time points. The role of this cytokine at the site of infection is not clear, but it likely supports neutrophil survival and function [30]. M-CSF, a growth factor for cells of the monocyte/macrophage lineage, is expressed only in the EP infected chambers, while this cytokine is not induced after *S*. *mitis* infection. Analysis of the response to the individual species suggests that *S*. *intermedius* is mainly responsible for the high level M-CSF response. Although we were unable to detect monocytes or macrophages in the recovered chamber cells, it is likely that tissue macrophages are associated with these infections, and that M-CSF acts to support their function [31]. A specific effect of *S*. *intermedius* on monocyte/macrophage function or expression is suggested by this result, but has not been previously reported. LIF (leukemia inhibitory factor) is similarly overexpressed in EP infection as compared to *S*. *mitis*. LIF is also induced by LPS [17] and is protective in bacterial pneumonia, where it protects from tissue damage and inhibits bacteremia [32]. It may provide a similar function in the chamber model of infection. Overexpression of LIF in EP infected chambers may also contribute to the lower levels of TNFα in these chambers up to 72 h, since LIF suppresses TNFα production [17].

## Conclusions

The cytokine and chemokine response to infection with the different organisms was strikingly different. Together, the results support the idea that the innate immune response to *S*. *mitis* is effective in attracting and supporting the function of cells that can kill and remove the bacteria. On the other hand, there are several defects in the innate response to the pathogenic species, likely reflecting multiple virulence mechanisms used by these species in persisting in the chamber infection. *P*. *intermedia* appears to be responsible for several of the cytokine responses elicited by the EP mixture, confirming that this species has a dominant effect on the host response in mixed infections. Further research will be needed to determine if manipulating the levels of one or several cytokines can be used to augment the host response to combat endodontic infections.

## References

[1] Siqueira JF Jr, Rocas IN. Diversity of endodontic microbiota revisited. J Dent Res. 2009;88(11):969–81. 10.1177/0022034509346549 . 19828883

[2] Marton IJ, Kiss C. Protective and destructive immune reactions in apical periodontitis. Oral Microbiol Immunol. 2000;15(3):139–50. 11154396 10.1034/j.1399-302x.2000.150301.x

[3] Iqbal MK, Kim S. A review of factors influencing treatment planning decisions of single-tooth implants versus preserving natural teeth with nonsurgical endodontic therapy. J Endod. 2008;34(5):519–29. 10.1016/j.joen.2008.01.002 . 18436028

[4] Stashenko P, Teles R, D'Souza R. Periapical inflammatory responses and their modulation. Crit RevOral BiolMed. 1998;9(4):498–521. 10.1177/10454411980090040701 9825224

[5] Sasaki H, Balto K, Kawashima N, Eastcott J, Hoshino K, Akira S, et al Gamma interferon (IFN-gamma) and IFN-gamma-inducing cytokines interleukin-12 (IL-12) and IL-18 do not augment infection-stimulated bone resorption in vivo. ClinDiagnLab Immunol. 2004;11(1):106–10. 10.1128/CDLI.11.1.106-110.2004 PMC321357 14715554

[6] Teles R, Wang CY, Stashenko P. Increased susceptibility of RAG-2 SCID mice to dissemination of endodontic infections. Infect Immun. 1997;65(9):3781–7. 9284152 10.1128/iai.65.9.3781-3787.1997 PMC175539

[7] Sasaki H, Hou L, Belani A, Wang CY, Uchiyama T, Muller R, et al IL-10, but not IL-4, suppresses infection-stimulated bone resorption in vivo. J Immunol. 2000;165(7):3626–30. 11034365 10.4049/jimmunol.165.7.3626

[8] Chae P, Im M, Gibson F, Jiang Y, Graves DT. Mice lacking monocyte chemoattractant protein 1 have enhanced susceptibility to an interstitial polymicrobial infection due to impaired monocyte recruitment. Infect Immun. 2002;70(6):3164–9. 12011011 10.1128/IAI.70.6.3164-3169.2002 PMC127982

[9] Rittling SR, Zetterberg C, Yagiz K, Skinner S, Suzuki N, Fujimura A, et al Protective role of osteopontin in endodontic infection. Immunology. 2010;129(1):105–14. 10.1111/j.1365-2567.2009.03159.x 19824920 PMC2807491

[10] Polak D, Wilensky A, Shapira L, Halabi A, Goldstein D, Weiss EI, et al Mouse model of experimental periodontitis induced by Porphyromonas gingivalis/Fusobacterium nucleatum infection: bone loss and host response. J Clin Periodontol. 2009;36(5):406–10. 10.1111/j.1600-051X.2009.01393.x . 19419440

[11] Matsui A, Jin JO, Johnston CD, Yamazaki H, Houri-Haddad Y, Rittling SR. Pathogenic bacterial species associated with endodontic infection evade innate immune control by disabling neutrophils. Infect Immun. 2014;82(10):4068–79. 10.1128/IAI.02256-14 . 25024367 PMC4187851

[12] Wilensky A, Polak D, Awawdi S, Halabi A, Shapira L, Houri-Haddad Y. Strain-dependent activation of the mouse immune response is correlated with Porphyromonas gingivalis-induced experimental periodontitis. J Clin Periodontol. 2009;36(11):915–21. 10.1111/j.1600-051X.2009.01464.x . 19735468

[13] Bland JM, Altman DG. Multiple significance tests: the Bonferroni method. BMJ. 1995;310(6973):170 7833759 10.1136/bmj.310.6973.170 PMC2548561

[14] Hou L, Sasaki H, Stashenko P. Toll-Like Receptor 4-Deficient Mice Have Reduced Bone Destruction following Mixed Anaerobic Infection. Infect Immun. 2000;68(8):4681–7. 10899873 10.1128/iai.68.8.4681-4687.2000 PMC98410

[15] Potempa M, Potempa J, Kantyka T, Nguyen KA, Wawrzonek K, Manandhar SP, et al Interpain A, a cysteine proteinase from Prevotella intermedia, inhibits complement by degrading complement factor C3. PLoS Pathog. 2009;5(2):e1000316 10.1371/journal.ppat.1000316 19247445 PMC2642729

[16] Byrne DP, Wawrzonek K, Jaworska A, Birss AJ, Potempa J, Smalley JW. Role of the cysteine protease interpain A of Prevotella intermedia in breakdown and release of haem from haemoglobin. Biochem J. 2010;425(1):257–64. 10.1042/BJ20090343 19814715 PMC2882103

[17] Ulich TR, Fann MJ, Patterson PH, Williams JH, Samal B, Del Castillo J, et al Intratracheal injection of LPS and cytokines. V. LPS induces expression of LIF and LIF inhibits acute inflammation. Am J Physiol. 1994;267(4 Pt 1):L442–6. . 7943346 10.1152/ajplung.1994.267.4.L442

[18] Hessle CC, Andersson B, Wold AE. Gram-positive and Gram-negative bacteria elicit different patterns of pro-inflammatory cytokines in human monocytes. Cytokine. 2005;30(6):311–8. 10.1016/j.cyto.2004.05.008 . 15935951

[19] Skovbjerg S, Martner A, Hynsjo L, Hessle C, Olsen I, Dewhirst FE, et al Gram-positive and gram-negative bacteria induce different patterns of cytokine production in human mononuclear cells irrespective of taxonomic relatedness. J Interferon Cytokine Res. 2010;30(1):23–32. 10.1089/jir.2009.0033 . 20028205

[20] Ye RD, Boulay F, Wang JM, Dahlgren C, Gerard C, Parmentier M, et al International Union of Basic and Clinical Pharmacology. LXXIII. Nomenclature for the formyl peptide receptor (FPR) family. Pharmacol Rev. 2009;61(2):119–61. 10.1124/pr.109.001578 19498085 PMC2745437

[21] Bizzarri C, Beccari AR, Bertini R, Cavicchia MR, Giorgini S, Allegretti M. ELR+ CXC chemokines and their receptors (CXC chemokine receptor 1 and CXC chemokine receptor 2) as new therapeutic targets. Pharmacol Ther. 2006;112(1):139–49. 10.1016/j.pharmthera.2006.04.002 . 16720046

[22] Herbold W, Maus R, Hahn I, Ding N, Srivastava M, Christman JW, et al Importance of CXC chemokine receptor 2 in alveolar neutrophil and exudate macrophage recruitment in response to pneumococcal lung infection. Infect Immun. 2010;78(6):2620–30. 10.1128/IAI.01169-09 20368349 PMC2876546

[23] Wuyts A, D'Haese A, Cremers V, Menten P, Lenaerts JP, De Loof A, et al NH2- and COOH-terminal truncations of murine granulocyte chemotactic protein-2 augment the in vitro and in vivo neutrophil chemotactic potency. J Immunol. 1999;163(11):6155–63. . 10570306

[24] AlShwaimi E, Berggreen E, Furusho H, Rossall JC, Dobeck J, Yoganathan S, et al IL-17 receptor A signaling is protective in infection-stimulated periapical bone destruction. J Immunol. 2013;191(4):1785–91. 10.4049/jimmunol.1202194 23863904 PMC3767040

[25] Balto K, Sasaki H, Stashenko P. Interleukin-6 deficiency increases inflammatory bone destruction. Infect Immun. 2001;69(2):744–50. 11159963 10.1128/IAI.69.2.744-750.2001 PMC97947

[26] Wang CY, Stashenko P. The role of interleukin-1 alpha in the pathogenesis of periapical bone destruction in a rat model system. Oral Microbiol Immunol. 1993;8(1):50–6. 8510985 10.1111/j.1399-302x.1993.tb00543.x

[27] Huang GT, Do M, Wingard M, Park JS, Chugal N. Effect of interleukin-6 deficiency on the formation of periapical lesions after pulp exposure in mice. Oral Surg Oral Med Oral Pathol Oral Radiol Endod. 2001;92(1):83–8. 11458250 10.1067/moe.2001.115025

[28] Biondo C, Mancuso G, Midiri A, Signorino G, Domina M, Lanza Cariccio V, et al Essential role of interleukin-1 signaling in host defenses against group B streptococcus. mBio. 2014;5(5):e01428–14. 10.1128/mBio.01428-14 25205091 PMC4166122

[29] Kawashima N, Stashenko P. Expression of bone-resorptive and regulatory cytokines in murine periapical inflammation. Arch Oral Biol. 1999;44(1):55–66. 10075151 10.1016/s0003-9969(98)00094-6

[30] Balamayooran G, Batra S, Theivanthiran B, Cai S, Pacher P, Jeyaseelan S. Intrapulmonary G-CSF rescues neutrophil recruitment to the lung and neutrophil release to blood in Gram-negative bacterial infection in MCP-1-/- mice. J Immunol. 2012;189(12):5849–59. 10.4049/jimmunol.1200585 23129755 PMC3518636

[31] Davies LC, Rosas M, Jenkins SJ, Liao CT, Scurr MJ, Brombacher F, et al Distinct bone marrow-derived and tissue-resident macrophage lineages proliferate at key stages during inflammation. Nature communications. 2013;4:1886 10.1038/ncomms2877 23695680 PMC3842019

[32] Quinton LJ, Mizgerd JP, Hilliard KL, Jones MR, Kwon CY, Allen E. Leukemia inhibitory factor signaling is required for lung protection during pneumonia. J Immunol. 2012;188(12):6300–8. 10.4049/jimmunol.1200256 22581855 PMC3370070

